# TIANA: transcription factors cooperativity inference analysis with neural attention

**DOI:** 10.1186/s12859-024-05852-0

**Published:** 2024-08-22

**Authors:** Rick Z. Li, Claudia Z. Han, Christopher K. Glass

**Affiliations:** Department of Cellular and Molecular Medicine, University of California, San Diego, La Jolla, CA 92093 USA

**Keywords:** Self-attention, Transcription Factors, Deep Learning, Integrated gradients, Bioinformatics

## Abstract

**Background:**

Growing evidence suggests that distal regulatory elements are essential for cellular function and states. The sequences within these distal elements, especially motifs for transcription factor binding, provide critical information about the underlying regulatory programs. However, cooperativities between transcription factors that recognize these motifs are nonlinear and multiplexed, rendering traditional modeling methods insufficient to capture the underlying mechanisms. Recent development of attention mechanism, which exhibit superior performance in capturing dependencies across input sequences, makes them well-suited to uncover and decipher intricate dependencies between regulatory elements.

**Result:**

We present Transcription factors cooperativity Inference Analysis with Neural Attention (TIANA), a deep learning framework that focuses on interpretability. In this study, we demonstrated that TIANA could discover biologically relevant insights into co-occurring pairs of transcription factor motifs. Compared with existing tools, TIANA showed superior interpretability and robust performance in identifying putative transcription factor cooperativities from co-occurring motifs.

**Conclusion:**

Our results suggest that TIANA can be an effective tool to decipher transcription factor cooperativities from distal sequence data. TIANA can be accessed through: https://github.com/rzzli/TIANA.

**Supplementary Information:**

The online version contains supplementary material available at 10.1186/s12859-024-05852-0.

## Background

In multicellular organisms, identical DNA is present in almost all cells, but diverse gene expression profiles lead to the development of various cell types and functions. *Cis*-regulatory elements (CREs) such as enhancers play a pivotal role in driving cell type-specific gene expression by orchestrating a complex interplay of multiple transcription factors (TFs) [1]. Enhancers serve essential functions in modulating gene expression profiles to suit specific cell types and states. These enhancer sequences harbor binding motifs for various TFs, and when the correct combinations of these factors converge and bind to the sequences, they initiate a series of events that result in the activation of enhancers and subsequently target genes [2, 3]. Combinatorial TF binding constitutes an intricate regulatory mechanism for enhancer selection and activation, ensuring that only the appropriate genes are activated within specific cell types and states [2, 4–6]. Consequently, only specific sets of enhancers containing binding motifs for the TFs needed for specific cellular states are activated. Notably, combinatorial cooperativities between TFs during enhancer selection and activation are highly dynamic, often contingent upon various environmental signals that alter TF activity and expression.

These cooperativities can result from different modes of DNA binding. For example, some TF pairs, such as GATA1 and TAL1, form complexes that that interact in a cooperative manner with composite recognition motifs exhibiting strict spacing relationships [7]. More often, combinations of TFs that exhibit interdependent binding relationships are observed at regulatory elements at which their corresponding DNA recognition motifs exhibit relaxed spacing relationships, such as macrophage lineage determining factors PU.1 and C/EBP family members [4, 7]. Most enhancer and promoter elements typically exhibit displacement of nucleosomes over a range of ~ 150 to 300 bp, suggesting that these regions can potentially be occupied by multiple factors relying on fixed and relaxed combinations of motifs. The combinatorial nature of enhancer regulation, involving the convergence of multiple TFs, underscores a unique opportunity to investigate regulatory mechanisms via enhancer sequence analysis.

Over the past decade, many state-of-the-art tools, such as Homer and the MEME suite, have been highly successful in identifying overrepresented TF motifs within regulatory sequences [4, 8]. While these tools provide critical insights into TF motif occurrence in regulatory sequences, they focus on individual motifs and are limited to inferring TF cooperation from CREs.

Recent advancements in deep learning, particularly convolutional neural networks and natural language processing (NLP) techniques, have shown promise in unraveling previously undiscovered regulatory mechanisms. Among them, multi-head attention (MHA) enables the model to simultaneously attend to different segments of the input sequence using multiple attention heads. This facilitates the learning of both short and long-range dependencies by allowing individual heads to capture local patterns and aggregate information across distant parts of the sequence. Moreover, through the parallel processing of multiple attention heads, MHA enables the learning of diverse representation subspaces. The versatility of MHA makes it an ideal method for studying genomic regulatory mechanisms, given the diverse ways regulatory elements interact. Notably, a recently developed deep learning model, SATORI, used MHA mechanisms to decipher TF cooperativities [9]. While the findings revealed significant cooperativities, several limitations need to be addressed. First, like most deep learning applications for genomic tasks, the initial convolution layer in SATORI model is designed to learn activation patterns. While this approach can learn de novo DNA motifs, annotating the convolution filters with motif libraries can be ambiguous and challenging [9, 10]. Additionally, NLP researchers have demonstrated that the attention scores produced by attention units, the approach employed in SATORI, exhibit limited interpretability and are susceptible to false cooperativities [11, 12]. Instead, NLP researchers show that saliency-based techniques such as integrated gradients can address this shortcoming for attention scores [11].

Here, we present Transcription factors cooperativity Inference Analysis with Neural Attention (TIANA), an MHA-based framework to infer combinatorial TF cooperativities from epigenomic data (ATAC-seq and ChIP-seq). To address the limitations of existing tools, TIANA uses known motif weights to initialize convolution filters to ease the interpretation challenge, allowing convolution filter activations to be directly associated with known TF motifs. In addition, TIANA uses integrated gradients to interpret the TF interdependencies from the attention units. We tested TIANA’s ability to recover TF co-binding pair motifs from ChIP-seq data, demonstrating that TIANA could identify key co-occurring TF motif pairs. We extended our validation to three lineage-determining TF (LDTF) ChIP-seq datasets and identified LDTF co-occurrences that are consist with previous literature findings. Last, we applied TIANA to the Kdo2-lipid A (KLA)-activated bone marrow-derived macrophage (BMDM) enhancer dataset, demonstrating TIANA can recover highly divergent functions associated with NFkB factors. The results suggest that TIANA is a highly robust tool that aims to assist in the identification of specific combinations of TFs that specify cell and signal-specific transcriptional outcomes.

## Implementation

### Overview of TIANA

**Fig. 1:**
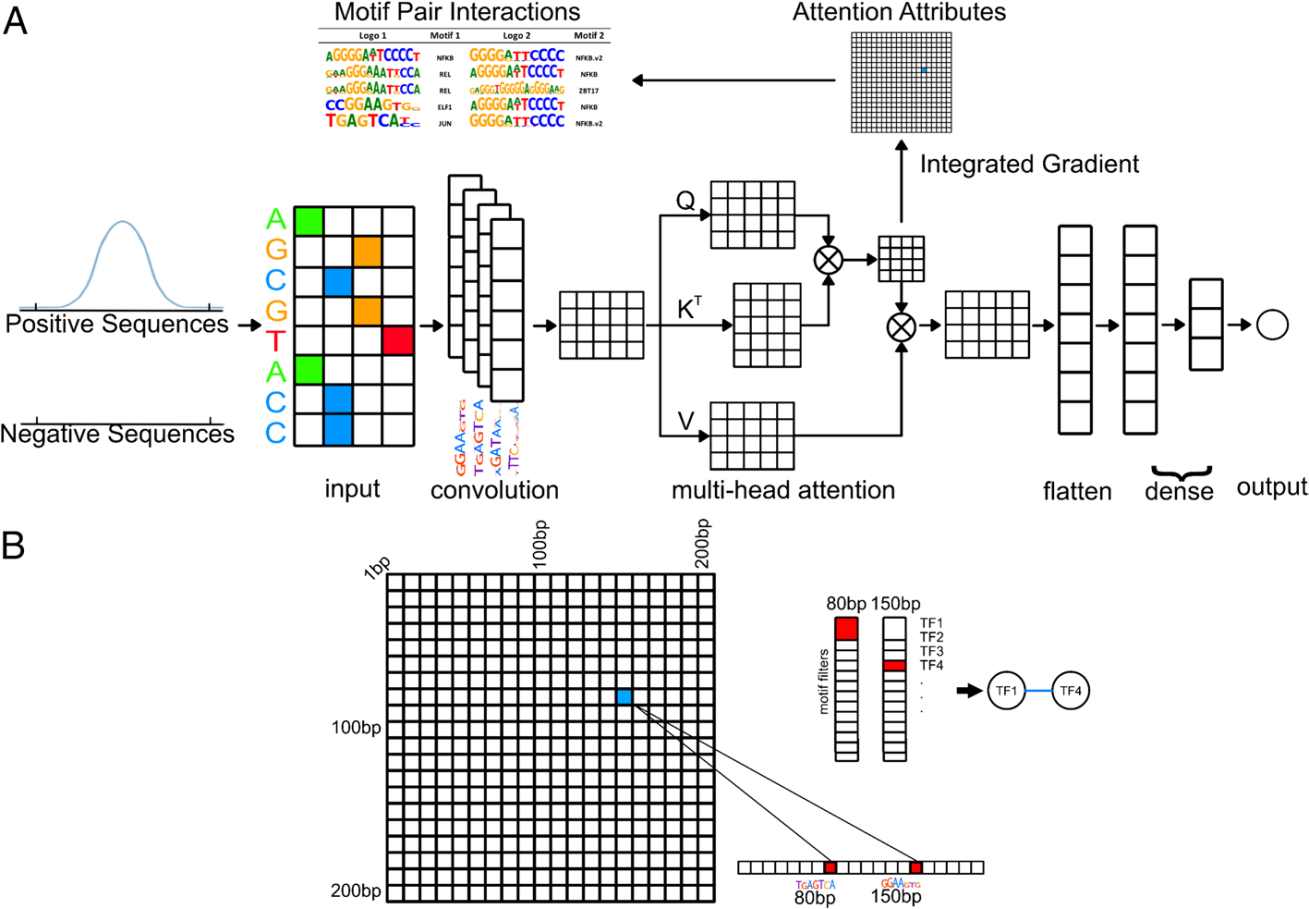
Overview of the TIANA implementation*.* **A** TIANA consists of two main steps. In the first Training Step, TIANA learns to classify one-hot encoded positive sequences from randomly generated negative sequences. Next, in the Interpretation Step, TIANA utilizes integrated gradient to compute attention attribute matrices from the MHA block, enabling the inference of co-occupancy of TFs. **B** In the Interpretation Step, active motifs are identified using motif score thresholds. As shown in the cartoon example in B, for a positive sequence, the TF1 motif ends at 80 bp, while the TF4 motif ends at 150 bp, colored in red. The attention attribute, highlighted in blue, connecting 80 bp and 150 bp, is used to associate two active motifs from these respective locations (shown as TF1 and TF4 are linked with a blue edge). For each motif pair, TIANA compares the attention levels between all positive sequences and negative sequences to determine whether the motif pair exhibits significantly higher attention in positive sequences than in random negative sequences

Figure 1A shows the overall model architecture and training strategy. TIANA consists of two primary steps: In the first step (denoted as Training Step), it employs a deep learning framework to classify between positive sequences (such as ATAC-seq and ChIP-seq peak sequences) and size-matched negative sequences (random genomic sequences). This classification step uses a neural network architecture that consists of one convolutional layer with preloaded known motif information and one self-attention layer. Attention heads allow the model to simultaneously focus on different aspects of the input sequence, enabling it to capture diverse patterns and relationships. In the second step (denoted as Interpretation Step), TIANA infers TF cooperativities using integrated gradients, which computes gradients on attention heads (denoted as attention attributes) of the fully trained model from the previous step. TIANA uses integrated gradients to quantify these cooperativities by first identifying active motifs from input sequences, next, TIANA links active motifs using the attention attribute. Lastly, TIANA compares attention attributes between positive and negative sequences to identify motif pairs that have significant attention levels to infer TF cooperativities. A schematic overview of required files to run TIANA can be found in Figure S1. Briefly, a peak file such as in bed format from a ChIP-seq/ATAC-seq experiment are required from the user. TIANA provides motif files as well as random genomic sequences used during training. The output of TIANA consists of a html report of significance levels of motif pairs, a corresponding csv file and a list containing all motif pairs identified and their corresponding attention attributes.

#### Architecture and training

TIANA was implemented using tensorflow v2.9.1. The first component of TIANA is a binary classification deep learning framework. TIANA takes training data consisting of positive sequences and negative sequences. Positive sequences are enhancers, open chromatin, or TF binding peak sequences. Negative sequences are noncoding random genomic sequences. Positive and negative sequences were one-hot encoded to A: [1,0,0,0];C: [0,1,0,0]: G: [0,0,1,0], T: [0,0,0,1]. All input sequences are 200 bp in size unless otherwise specified, and TIANA can tolerate input sizes up to 1000 bp. For data used in this study, chr1, chr8, and chr18 were held out as the evaluation testing set; other chromosomes were used for training. Training performance of dataset used in this study are listed in Supplementary Table 1.

The first layer of the TIANA model is a one-dimensional convolution layer that uses filters to identify short nucleotide patterns. The convolution layer is preloaded with known motif position-specific score matrices (PSSMs) and set to be fixed. We obtained known motifs from JASPAR motif library [13, 14]. To reduce the redundancy of motifs, we used a hierarchical clustering function from TBA to group highly similar motifs [15]. The *Biopython motif* module was used to convert Jaspar-format motifs to motif PSSMs, which were loaded into convolution layers and computed the motif score cutoff. Unless otherwise specified, the convolution layer used in this study consists of 448 filters, with 224 motifs and their reverse complement motifs.

We used a vertical 1D max-pooling layer with a pooling size of 2, after the convolution layers to capture the highest activation score for each forward-reverse complement motif pair. By default, TIANA combines highest forward and reverse complement motif scores at each position. TIANA also provides an optional approach that computes forward and reverse complement motif separately through raising the *–compute_rc* flag when calling *tiana.py*. The vertical max-pooling layer was followed by a batch normalization layer and a 1D horizontal max-pooling layer, with pool size of 4 to reduce the computational overhead and improve model generalizability [16]. A detailed TIANA model summary can be found in Supplementary Table 2. For the examples used in this study, TIANA was trained for 50 epochs.

#### Positional encoding

We used a positional encoding method based on sine and cosine functions to preserve the positional information [17]. Suppose that the input from a previous layer is $X_{{{\text{input}}}} \in R^{{m \times f}}$ where $m$ is the sequence size and $f$ is the total number of motifs/filters. The positional encoding matrix $P\in {R}^{m\times f}$ shares the same dimension as ${X}_ {{\text{input}}}$. For each element ${P}_{i,2j}$ or ${P}_{i,2j+1}$, the positional embedding values are computed as:

$${P}_{i,2j}=sin\left(\frac{i}{{n}^{2j/f}}\right)$$

$${P}_{i,2j+1}=cos\left(\frac{i}{{n}^{2j/f}}\right)$$

where n is a scalar factor with a default value of 10,000. The output matrix can be written as ${X}_{out\text{put}}={X}_{in\text{put}}+P$.

#### Multi-head attention unit

The output of positional encoding was then fed into the multi-head attention block, which consisted of Query ($Q$), Key (k) and Value ($V$) matrices. The QKV matrices provide the framework for the attention mechanism, with the Query matrix generating queries, the Key matrix providing contextual information, and the Value matrix holding the actual content, collectively facilitating effective attention computation and learning. TIANA uses one MHA layer with four attention heads. For each attention head $h$, the score matrix ${S}_{h}\in {R}^{m\times m}$ is computed using:

$$Attention\left({S}_{h}\right)=softmax\left(\frac{Q{K}^{T}}{\sqrt{{d}^{k}}}\right)$$

where: ${d}_{k}$ represents the size of Key (k).

The output of the MHA unit is computed by taking the dot-product of attention score matrix $S$ with value matrix $V$:

$$A=S \times V$$

The output of the MHA unit was then fed into a flattened layer, as well as two fully connected layers with 256 and 64 units, respectively. The output of the fully connected layers was then fed into a single unit output layer with a sigmoid activation function for binary classification.

#### Integrated gradients

We used integrated gradients of the attention matrix $S$ to measure the TF cooperativities. We denote $G\in {R}^{m\times m}$ as the integrated gradients attention attribute matrix for each attention score matrix $S$. Using the methodologies described previously [11, 18], $G$ can be approximated as:

$${\text{G}} = \frac{{\text{S}}}{{\text{a}}} \odot \sum _{{{\text{k}} = 1}}^{{\text{a}}} \frac{{\partial {\text{F}}\left( {\frac{{\text{k}}}{{\text{a}}}{\text{S}}} \right)}}{{\partial {\text{S}}}}$$

where, $a$: number of steps in the Riemann approximation with default value of 100. $F$: TIANA’s classification function. $\frac{\partial F}{\partial S}$: gradient of F relative to attention score matrix S. $\odot$: elementwise multiplication.

### Quantifying transcription factor cooperativities

Figure 1B provides a schematic of the process to quantify TF motif cooperativities after the Training Step. First, for each sequence, active motifs are identified by having activation values from convolution layers higher than motif score thresholds computed previously using *Biopython motif* module with a *p*-value threshold of 1e−4. The positional information of activated motifs was preserved in attention attribute matrices $G$. Next, for each sequence, TIANA computes max attention attributes by calculating the position-wise maximum over the attention attribute matrices across all attention heads to produce ${G}^{max}\in {R}^{m\times m}$ matrix.

The max attention attribute value ${G}_{i,j}^{max}$ was used to link two active motifs at two positions $i$ and $j$ for ${\forall }_{i}\in \left\{\text{1,2},\dots m\right\}$ and ${\forall }_{j}\in \left\{\text{1,2},\dots m\right\}$. In the cartoon example shown in Fig. 1B, TF1 and TF4 motifs are active in position 80 bp and 150 bp, thus TF1 and TF4 are linked using the attention attribute value ${G}_{\text{80,150}}^{max}$ (blue edge connecting TF1 and TF4 in Fig. 1B). For each sequence, if a TF motif is active at multiple locations, thus a motif pair has multiple attention attributes, the highest attribute value was used for the next significance testing step. Summary statistics, including number of peaks used in each dataset and number of motif pairs with attention attributes are listed in Supplementary Table 3.

### Identifying significant motif pairs

For each pair TF motif, we compare the attention attributes for all the peaks between positive and negative sequences. To eliminate noise from low-quality predictions, positive peaks with sigmoid activation less than 0.7 and negative peaks with activation higher than 0.3 from the Training Step were removed from the significance testing. We used Mann–Whitney U test to compare the significance of attention attributes differences of a TF pair in positive and negative sequences. All *p*-values were corrected using Bonferroni correction method. The output of TIANA reports all significant (*p*-values < 1e−4) TF pairs from the positive sequences, sorted by *p*-values.

### Sequencing data processing

ATAC-seq and H3K27ac ChIP-seq for KLA activated BMDM were downloaded from GEO (accession: GSE109965) [19]. LDTF ChIP-seq data for ATF3, SPI1, and CEBPB data were downloaded from GEO (accession: GSE111856 and GSE109965) [15, 19]. ETS1 and RUNX1 co-binding data were downloaded according to Martin et al. [20] from ENCODE (ID: ENCSR588AKU for RUNX1 and ID: ENCSR000BKQ for ETS1). Oct4/Sox2 ChIP-seq data were downloaded from Malik et al. [21] (accession: GSE103980). TF co-binding ChIP-seq data were obtained from the ENCODE data portal, as described in Shen et al. [7]. Sequencing reads were mapped using *Bowtie2*, using either human GRCh38/hg38 or mouse GRCm38/mm10 as a reference genome [22]. We used *Homer findPeaks* to identify peaks and used *IDR* to obtain reproducible peaks [23]. Peaks were resized to 200 bp and converted to a bed format before converting into one-hot encoding formats for training and testing with TIANA.

Enhancers were called from ATAC-seq and H3K27ac ChIP-seq using a similar method described previously [24]. First, we identified reproducible open chromatin peaks using ATAC-seq data. Next, we used *Homer annotatepeaks.pl* function to identify peaks with > 16 H3K27ac tag counts. Finally, we selected regions with > 2.5 fold change in H327ac, marked as “KLA-activated” and “KLA-repressed” peaks.

### Comparative methods

We compared TIANA to SATORI, another MHA-based model used to infer TF cooperativities. We followed the method described in SATORI to extract the attention score matrix, which we compared with the attention attribute matrix from TIANA. Since the attention score (SATORI) and attention attributes (TIANA) cannot be directly compared due to a different data distribution, we computed rank for the attention score and attention attributes for comparison.

## Results

### TIANA prediction performance

**Fig. 2:**
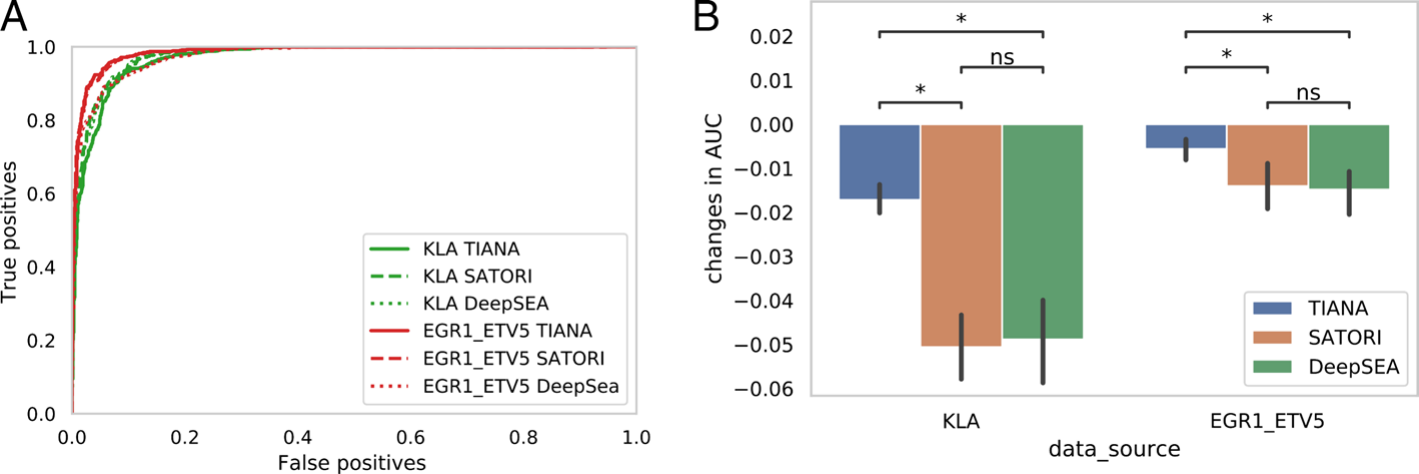
Comparisons of classification performance using SATORI, DeepSEA and TIANA*.* **A** ROC curves showing the performance of SATORI, DeepSEA and TIANA on KLA activated enhancers in BMDMs (denoted as KLA), and EGR1_ETV5 co-binding peaks in K562 cells (denoted as EGR1_ETV5). Solid, dashed and dotted lines represent the performances of TIANA, SATORI and DeepSEA, respectively. **B** Changes in AUC scores when using 25% of the original training peaks. Random down sampling was performed five times in each model/sample and compared to the performance of the full model in A. Error bars represent 95% confidence intervals

We compared the classification performance of TIANA with two other deep learning models, SATORI and DeepSEA. Both TIANA and SATORI utilize convolution and multi-head attention units in their architectures, while DeepSEA employs convolutional layers in its network. Figure 2A shows the receiver operating characteristic (ROC) curves for TIANA, SATORI, and DeepSEA. In KLA-activated BMDM enhancer peaks and EGR1_ETV5 co-binding ChIP-seq peaks in K562 cells, all three models performed well in classification, with area under the ROC curve (AUC) scores exceeding 0.9.

Next, because assays such as ChIP-seq and ATAC-seq typically yield a wide range of peaks, ranging from a few thousand to tens of thousands in different cell types and conditions, it is necessary to evaluate the robustness of TIANA’s learning performance, especially with a low number of input peaks. Thus, we retrained TIANA, SATORI, and DeepSEA models with only 25% of the original training sequences through random down sampling, while using the same number of peaks for testing (all peaks in chr1, 8, and 18) and compared the change in performance in these models. As seen in Fig. 2B, SATORI and DeepSEA tend to exhibit a greater decrease in classification performance when trained with only 25% of the original input sequences.

We reason that SATORI and DeepSEA require sufficient input sequences to learn convolution filters that represent motifs, while TIANA only needs to learn TF cooperativities with preloaded motif weights. The comparison of training performance supports the notion that TIANA’s approach, which preloads motif weights, has a minimal impact on overall learning performance while offering robust learning performance with limited input data.

### Identification of TF cooperativities in embedded motifs

To benchmark TIANA, we conducted simulation experiments to embed three motif pairs, SPI1-SIX1, SPI1-CEBPE, and RXRA-NRF1, into genomic sequences using similar approaches as previously published [9, 25]. In summary, we randomly selected 40,000 DNA sequences and embedded one pair of motifs (referred to as Motif 1 and Motif 2) into 20,000 sequences, which we denote as random sequences. For the remaining 20,000 sequences, which served as negative sequences, we embedded either Motif 1 or Motif 2, but not both, in each sequence. We repeated this simulation five times for each of the motif pairs.

Among all the embedded motif pairs, TIANA successfully identified these pairs as significant motif pairs with *p*-values equal to or close to zero with close to 100% classification performance. The results of the simulation experiment are summarized in Supplementary Table 4.

### TIANA identifies of TFs for collaborative binding

TAL1 and GATA1 are a well-documented TF pair that dimerize upon binding to a WGATTA (GATA1) and a partial E-box (TAL1) motifs located 7–9 bp apart. Prior experimental investigations also suggest that GATA1-TAL1, instead of any other TFs, have a pioneering role in setting up co-binding peaks and subsequent cellular functions [26]. Considering the experimental findings, it is reasonable to hypothesize that a robust computational model will identify TAL1 and GATA1 motifs, especially when located between 7 and 9 bp, as the principal determinant for GATA1-TAL1 co-binding. We thus obtained previously identified co-binding peaks from a K562 cell line and compared the performance of TIANA and SATORI to recover TAL1-GATA1 co-occurrences and corresponding attention levels. Table 1 summarizes the significance level and motif pairs recovered from both models.

**Table 1:** *p*-values of the GATA1-TAL1 motif recovered from GATA1-TAL1 ChIP-seq co-binding peaks and percent of peaks with active GATA1-TAL1 motif filters located 7–9 bp apart

Model	Adjusted *p*-values	Peak with active GATA1-TAL1 filters 7–9 bp (%)
TIANA	0.0	68.1
SATORI	3.5e−26	7.9

**Fig. 3:**
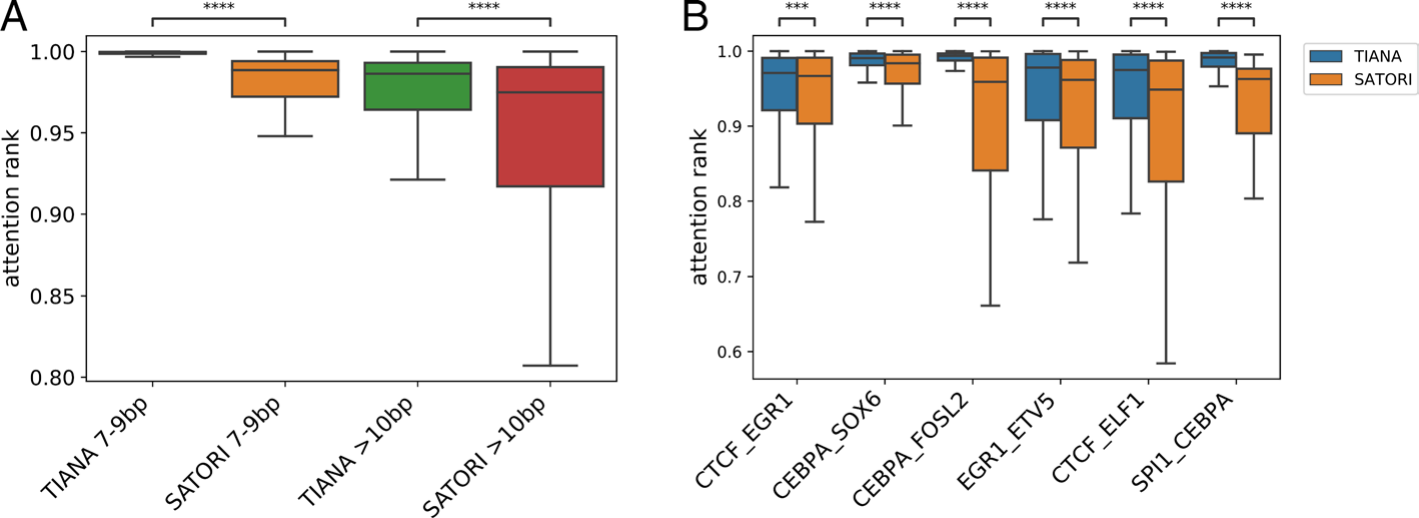
Attention level comparison of TIANA and SATORI on TF co-binding motifs*.* **A** Attention levels for GATA1-TAL1 co-binding peaks, motifs located between 7 and 9 bp and > 10 bp are shown separately. **B** Attention levels for six pairs of TFs that are known to have relaxed spacing relationships. (Mann–Whitney U test, ***: 4.00e−09 < *p* <  = 4.00e−08; ****: *p* <  = 4.00e−09)

Although both methods can successfully identify GATA1-TAL1 motif pairs as highly significant pairs, SATORI identified 8% of GATA1-TAL1 co-binding peaks with corresponding motif pairs located between 7 and 9 bp. In contrast, TIANA’s motif pair identified 68% of co-binding peaks containing GATA1-TAL1 motifs. TIANA’s finding confirms previous results, which suggested that three-quarters of co-binding peaks bound by GATA1-TAL1 were located 7–9 bp apart [26]. Figure 3A shows attention levels for motif pairs located between 7 and 9 bp and > 10 bp for both models. TIANA demonstrated higher levels of attention to TAL1-GATA1 in both constrained spacing (7–9 bp) and > 10 bp compared with SATORI. Comparing with the preload motif approach employed by TIANA, the convolution layer in SATORI identified multiple filters that are all partially matched to the consensus TAL1 half E-box motif, consistent with the previous reports [9, 26].

To further validate if TIANA can identify motif-orientation specific TF pairs, we analyzed the results from reverse complement-enabled outputs on GATA1-TAL1 co-binding peaks. As seen in Figure S2A, for GATA1-TAL1 motifs, TIANA predicts TAL1 upstream of GATA1 (or downstream if both motifs are reversed) with high attention levels, this result is consistent with prior experimental data showing TAL1 motif (half E-box) frequently locates 7–9 bp upstream of GATA motif (WGATAA) [26]. Similarly, we applied TIANA to ETS1-RUNX1 co-binding peaks used in Martin et al., [20]. As shown in Figure S2B, similar levels of attention attributes are observed for different orientations as well as the spacings between motifs. The attention attributes identified here also align with the experimental findings from Martin et al. [20]. We also applied TIANA to SOX2-OCT4 motif pairs, a well-characterized TF pair that frequently heterodimerize to perform essential biological functions [21, 27–29]. As shown in Figure S2C, similar to TAL1-GATA1, SOX2-OCT4 exhibits orientation and position preference, consistent with prior findings [21, 27].

Next, we compared the attention performance between TIANA and SATORI with TF pairs that are known to co-bind with relaxed spacing relationships [7]. We obtained the co-binding peaks for each TF pair dataset and compared the attention levels, attention attributes from TIANA and attention scores from SATORI, that matched the corresponding motifs. Figure 3B shows the attention rank between TIANA and SATORI, where TIANA shows a consistently higher attention level than SATORI. Although other TF motifs may influence the co-binding of these TF pairs, it is reasonable to believe these motif pairs play the primary role in the co-binding peaks. This finding further demonstrates that TIANA has a superior ability to identify important TF cooperativities compared to the existing tool.

### Discoveries of motif dependencies in lineage-determining factors

**Fig. 4:**
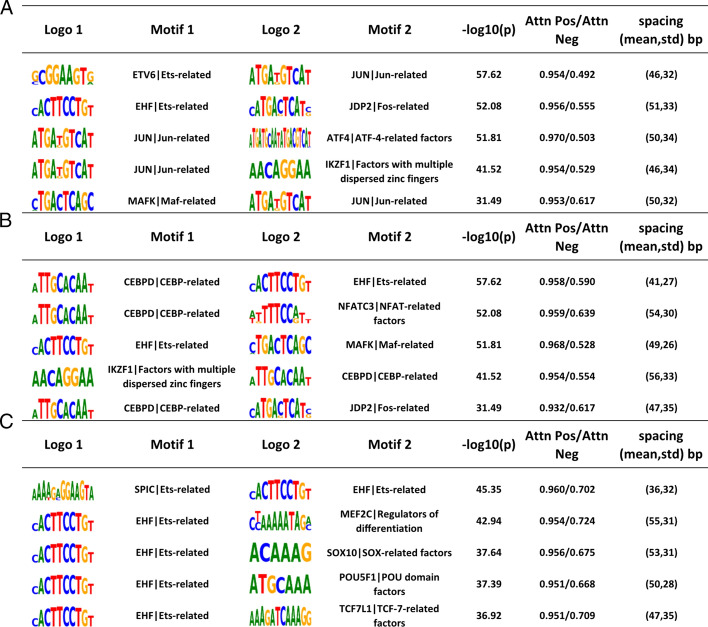
Top TF motif pair identified from LDTF ChIP-seq data*.* **A** ATF3 **B** CEBPB and **C** SPI1 ChIP-seq ranked by *p*-values. The last three columns show the − log10 of the *p*-values for each motif pairs, mean attention levels in positive sequences verses negative sequence, and summary statistics (mean, standard deviation) of the spacing relationship between the motif pairs in positive sequences, respectively

LDTFs are TFs that are required to establish epigenetic landscape for cell lineages, some of which have chromatin opening abilities attributed to pioneer factors. Commonly known LDTFs for macrophages include SPI1, CEBP family, and AP-1 factors. Thus, we obtained ChIP-seq data for SPI1, CEBPB, and ATF3 (AP-1 family) and used TIANA to identify the top motif pairs for each LDTF. Figure 4 shows the top 5 results for each ChIP-seq dataset. The result for PU.1 ChIP-seq suggests that PU.1 largely depends on its own SPI1/Ets-factor motifs, as evidenced by all other motifs needing to pair with an SPIC or other Ets-factor motifs. This observation is consistent with the well-established knowledge that SPI1 acts as a potent chromatin modifier as it is the master regulator for the myeloid lineage that establishes the chromatin landscape, allowing other LDTFs to bind [30]. The ATF3 ChIP-seq results show that the AP-1 family such as Fos and Jun related motifs are strongly associated with the Ets motifs. Last, the results for the CEBPB ChIP-seq suggest that the CEBPB motif is strongly associated with SPI1/Ets motif and MEF2C motif. These findings are consistent with prior findings and confirm TIANA’s ability to identify biologically relevant information [24].

### Divergent roles of NF${\varvec{k}}$B in macrophage enhancers

**Fig. 5:**
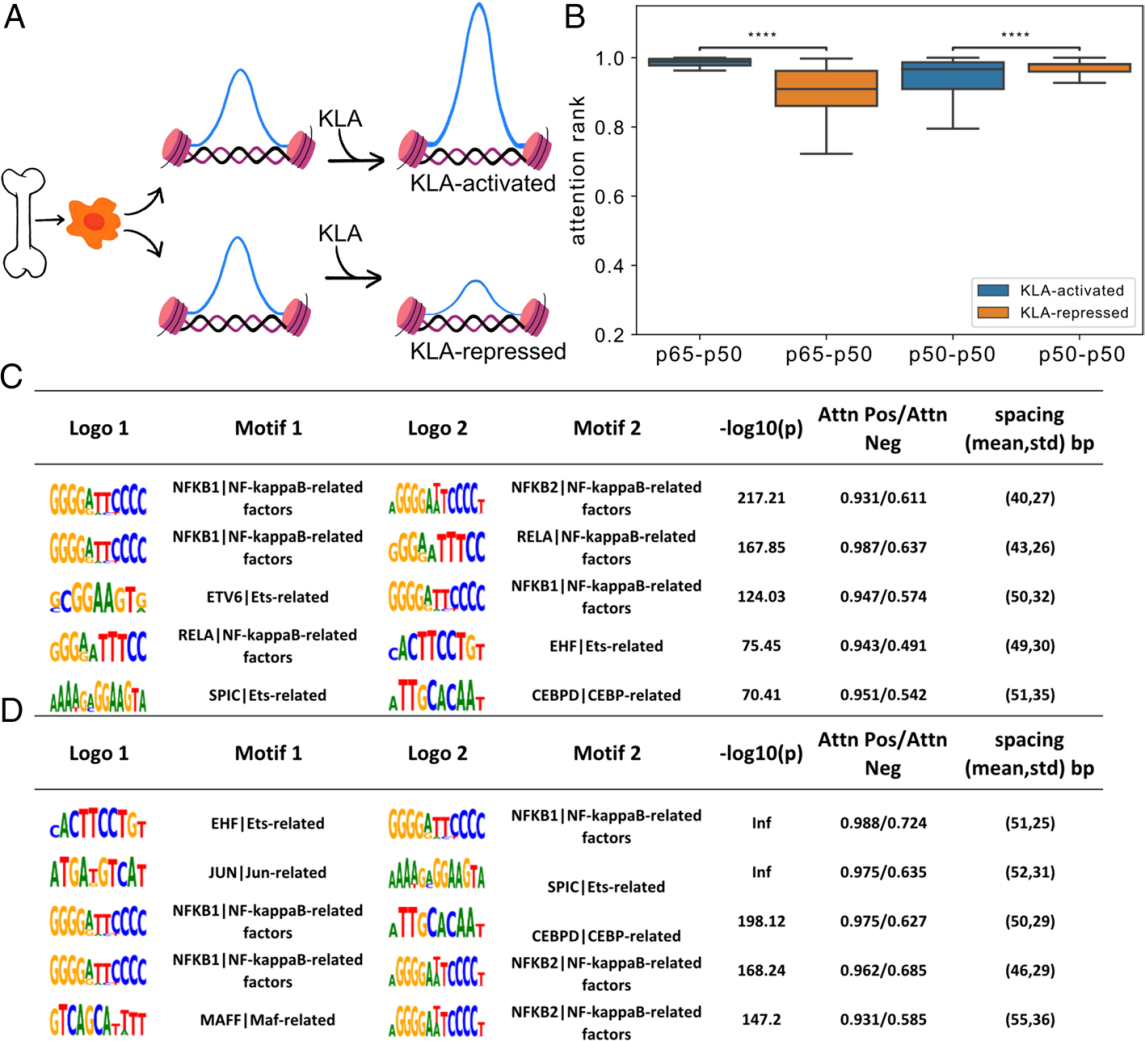
TIANA identifies divergent NF-kB factor functions in macrophage regulatory elements*.* **A** Schematics showing KLA-activated and repressed enhancer regions in bone-marrow-derived macrophages. **B** Boxplot showing attention attribute levels for p65–50 heterodimer motifs and p50-p50 homodimer motifs in KLA-activated and repressed enhancers. (Mann–Whitney U test, ****: *p* <  = 4.00e−09) **C** Similar to Fig. 4, top TF motif pairs identified in KLA-activated enhancers. **D** Top TF motif pairs identified in KLA-repressed enhancers

Finally, we evaluated TIANA’s performance with differentially activated and repressed enhancers in KLA-stimulated BMDMs. As shown in the schematics in Fig. 5A, the addition of substrate KLA will activate and repress different sets of enhancers in BMDMs. Figure 5B demonstrates that TIANA assigned significantly higher attention to the REL (p65)-NFkB (p50) heterodimer motif pair in KLA-activated enhancers than in KLA-repressed enhancers. On the other hand, KLA-repressed enhancers show greater attention to the p50–p50 homodimer than the KLA-activated enhancers. Figure 5C and Fig. 5D show the top significant motif pairs TIANA identified. In addition to the divergent roles of NFkB factors in which heterodimers are preferred in KLA activated enhancers and homodimers are dominant in HLA-repressed enhancers, TIANA also identifies several key motifs associated with each group that is known to associate with KLA-stimulated and repressed macrophages, such as SPI1 (SPI1/Ets) and AP-1 (MAFF and JUN) [24, 31].

This finding is consistent with previous experimental data showing that p65-p50 heterodimers are preferentially associated with KLA-activated enhancers, whereas p50 homodimerizes in KLA-repressed enhancers [32–34]. Due to motif similarities between REL/p50 and NFκB/p65, traditional motif enrichment tools cannot identify such divergent functions [24]. Existing tools, such as MAGGIE, with sufficient resolution to assign such divergent roles require multiple strains of mouse data to leverage genomic variant information [24], while TIANA can obtain similar findings using data from one strain of mouse without additional strain variant information.

## Discussion

This study demonstrates that TIANA is a robust tool to infer cooperative TF co-occurrence from epigenomic data. TIANA was built to improve interpretation, as we recognize that interoperability can be a significant obstacle for deep learning-based tools’ applications in genomics. We preloaded known motifs as convolution filter weights. Although this may limit the kind of DNA patterns that can be learned in the training stage, we show that the “known motif” approach employed by TIANA is still able to achieve satisfactory learning performance compared with similar methods such as DeepSEA and SATORI. Moreover, we also demonstrated that the "known motif" approach could tolerate various input sequence numbers without suffering significant performance drops.

Interestingly, our data suggest that commonly used "learned motif" approach, used by existing tools such as SATORI and DeepSEA, may have a limited ability to tolerate differences in motif sizes. To the best of our knowledge, there has yet to be a consensus on the appropriate filter size used in convolution layers. Currently, the commonly used convolution filter sizes can range from 5 to 20 bp [9, 35–37]. However, TF motifs are naturally very different in size. For example, an experimentally validated dataset suggested that the TAL1/half E-box motif can be as short as 3–4 bp, while CTCF motif is 19 bp [26, 38]. Having long motif filters in the architecture unavoidably allows other unwanted patterns to be learned/activated. On the other hand, short motif filters will lead to ambiguity, since many distinct TFs, at least in part, share very similar patterns. Annotating filters to motifs has been frequently reported as a challenge to fully interpret deep learning models used in genomics [9]. Using the TAL1 motif as an example, the learned filter by SATORI contains patterns much longer than the consensus motif. It is reasonable to believe that suboptimal filters will burden the annotation of filters with known motifs and limit the interpretability of attention scores/attributes, since accurate motifs/filters will lead to flawed attention between features.

The improved ability of TIANA to identify TF co-occurrence can also be attributed to the integrated gradients/saliency-based method employed in the model. Given that saliency-based methods have become an essential interpretation technique and are critical to understanding the feature contribution in machine deep learning models [18, 39], TIANA demonstrates how it can also assist in the discovery of biologically relevant insights.

Our application of TIANA to LDTF ChIP-seq and KLA-activated BMDM enhancers showed that TIANA can recover insights that were previously only available with experimental validations or tools that leverage genomic variant information, such as MAGGIE. It should be noted that TIANA does not leverage genomic variants and, thus, can be adapted to a wide range of datasets with simple experimental setups. Moreover, unlike many existing tools that focus on discovering individual motifs/TFs, TIANA focuses on inferring significant TF cooperativities pairs among the co-occurring motifs from the input sequences. This is an advantage of TIANA because TF interdependency and/or exclusivity were largely unaccounted for previously. Collectively, we demonstrated that TIANA is an effective tool to investigate regulatory mechanisms hidden in regulatory sequences.

## Conclusion

Sequences within regulatory elements often contain rich mechanistic insights into cell-type specific functions. Deep learning-based tools can be highly effective at deciphering the hidden information within these sequences if the model can be interpreted. We demonstrated that TIANA is an effective interpretation-oriented deep learning tool to infer hidden TF cooperativities information embedded within nucleotides.

### Supplementary Information

Supplementary Material 1.

## Data Availability

ATAC-seq and H3K27ac ChIP-seq for KLA activated BMDM were downloaded from GEO (accession: GSE109965) [19]. LDTF ChIP-seq data for ATF3, SPI1, and CEBPB data were downloaded from GEO (accession: GSE111856 and GSE109965) [15, 19]. TF co-binding ChIP-seq data were obtained from the ENCODE data portal, as described in Shen et al. [7]. DNA motif library used in this study is available at JASPAR motif database at https://jaspar.elixir.no/downloads/. The code for TIANA is available from its Github repository at https://github.com/rzzli/TIANA.

## Declarations

### Availability and requirements

Project name: TIANA. Project home page: https://github.com/rzzli/TIANA. Operating system(s): Platform independent. Programming language: Python. Other requirements: Python 3.8, Tensorflow 2.9.1, Keras 2.9.0, NumPy 1.22 or higher, Pandas 1.2.4 or higher, Matplotlib 3.3.2 or higher, Seaborn 0.12.2 or higher, Scikit-learn. License: MIT License. Any restrictions to use by non-academics: No restrictions.
