# Supplementary material for: TIANA: transcription factors cooperativity inference analysis with neural attention
Source: BMC Bioinformatics. 2024 Aug 22;25:274. doi: 10.1186/s12859-024-05852-0 (PMC11342676; doi:10.1186/s12859-024-05852-0)
Supplement: Supplementary file 1 — Supplementary Material 1. [file 12859_2024_5852_MOESM1_ESM.docx]

# Supplemental Information


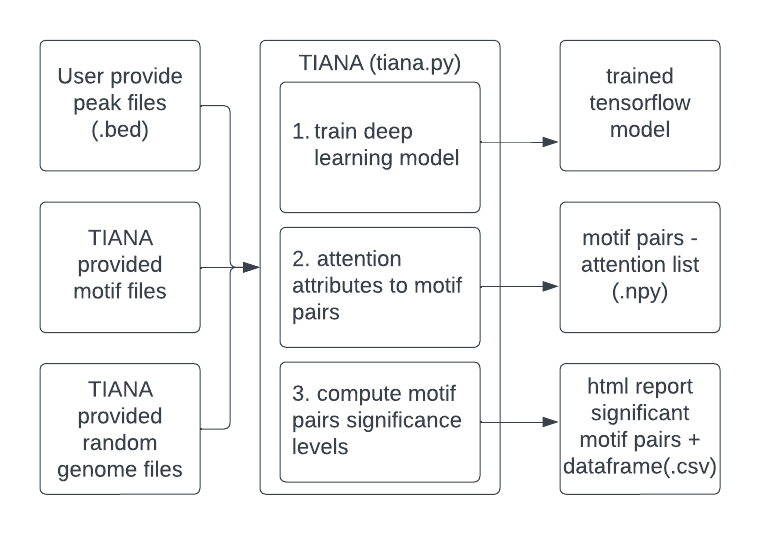


**Figure S1 Schematics of TIANA workflow**

TIANA requires users to input a peak file, such as bed format. TIANA will take the input peak sequences, together with TIANA predefined random genomic sequences as negative sequences (currently only hg38 and mm10 are supported); to first train the deep learning model. After the training, TIANA will next compute motif pairs with attention attributes for each sequence. Lastly, TIANA will compare the attention attributes level between positive (user provided peaks) and negative (random genomic) sequences, and compute significance level for motif pairs and generate html report and corresponding csv file specifying motif pairs with significance levels.


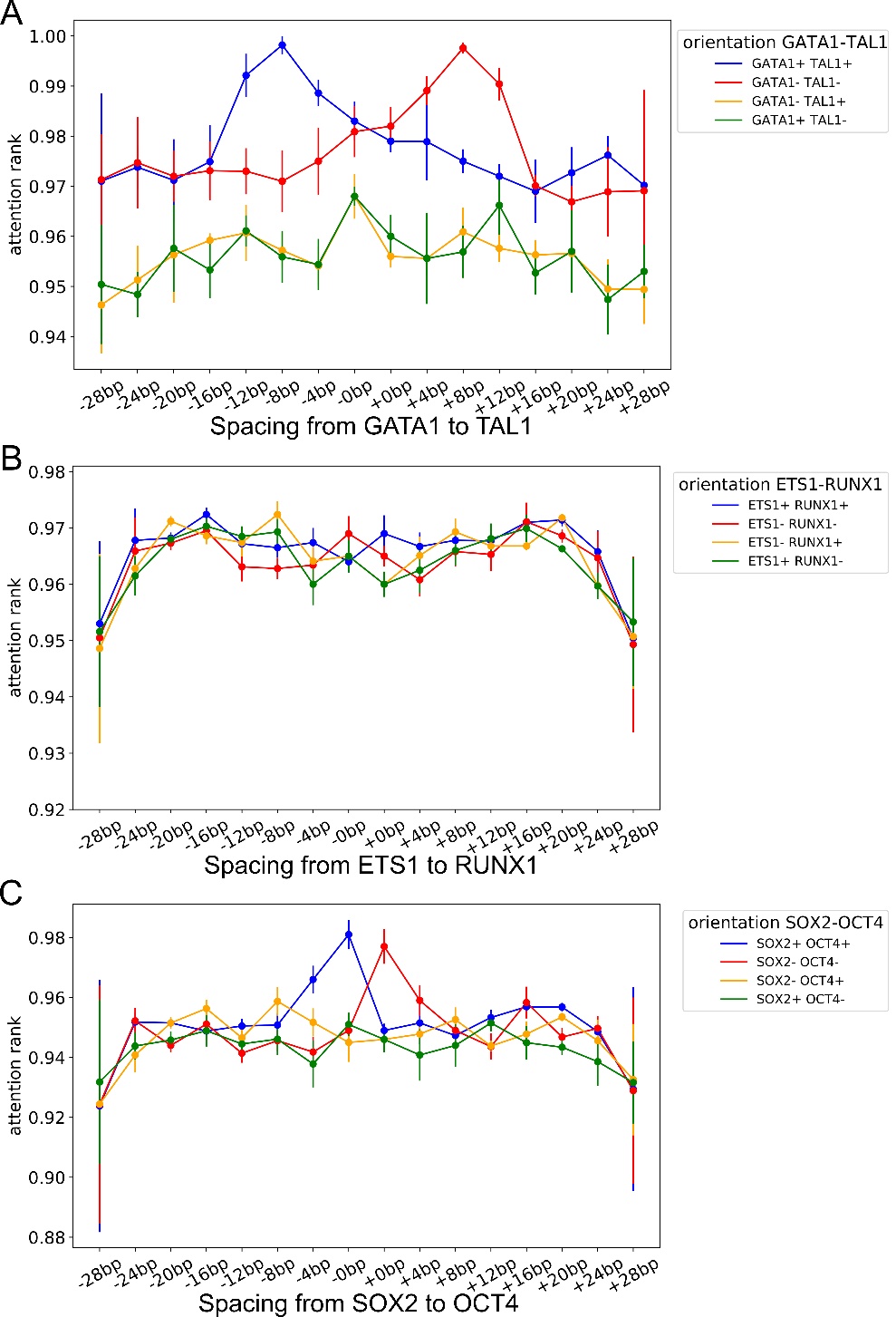


**Figure S2 TIANA differentiates orientation-specific preference in transcription factor pairs.**

A) The attention levels derived from GATA1 and TAL1 co-binding peaks across various motif orientations and positional relationships. Forward-oriented motifs are denoted by GATA1+ and TAL1+, while reverse complement orientations are indicated by GATA1- and TAL1-. The X-axis represents the positional relationship between the two motifs, where "-[x] bp" represents TAL1 positioned upstream of GATA1 and "+[x] bp" signifies the opposite. Each line and point are colored to represent different combinations of motif orientations. The red and blue lines represent same motif orientation combinations (GATA1+TAL1+/GATA1-TAL1-) found on opposite DNA strands, same for the yellow and green lines (GATA1+TAL1-/GATA1-TAL1+). Points depict the average attention rank (Y-axis) for motif pairs within specific spacing distances (grouped into 4bp bins) and their corresponding orientation combinations. Error bars represent the standard deviation of the attention rank. B) and C) attention levels for ETS1-RUNX1 and SOX2-OCT4 motif pairs, respectively, similar to panel A.

Supplementary Table 1 Classification performance of data for Figure 3-5

| Dataset | AUC | PRC |
| --- | --- | --- |
| EGR1_ETV5 | 0.945 | 0.934 |
| GATA1_TAL1 | 0.962 | 0.951 |
| CECF_EGR1 | 0.912 | 0.921 |
| CEBPA_SOX6 | 0.91 | 0.90 |
| CTCF_ELF1 | 0.951 | 0.979 |
| ETS1_RUNX1 | 0.912 | 0.89 |
| CEBPA_FOSL2 | 0.98 | 0.961 |
| ATF3 | 0.962 | 0.973 |
| CEBP-beta | 0.902 | 0.910 |
| PU1 | 0.942 | 0.93 |
| KLA activated BMDM enhancer | 0.974 | 0.984 |
| KLA repressed BMDM enhancer | 0.995 | 0.99 |

Supplementary Table 2 TIANA model summary

| Layer Type | Output Shape | Param # |
| --- | --- | --- |
| InputLayer | (None, 200, 4) | 0 |
| Conv1D | (None, 179, 448) | 39424 |
| myMaxPool | (None, 179, 224) | 0 |
| BatchNormalization | (None, 179, 224) | 896 |
| Activation | (None, 179, 224) | 0 |
| MaxPooling1D | (None, 45, 224) | 0 |
| Dropout | (None, 45, 224) | 0 |
| PositionalEncoding | (None, 45, 224) | 0 |
| AttentionBlock | ((None, 45, 224),(None, 4, 45, 45),(None, 45, 4, 56)) | 404096 |
| Flatten | (None, 10080) | 0 |
| Dropout | (None, 10080) | 0 |
| Dense | (None, 256) | 3E+06 |
| Activation | (None, 256) | 0 |
| Dropout | (None, 256) | 0 |
| Dense | (None, 64) | 16448 |
| Activation | (None, 64) | 0 |
| Dense | (None, 1) | 65 |
| Total params: 3,041,665 | | |
| Trainable params: 3,000,897 | | |
| Non-trainable params: 40,768 | | |

Supplementary Table 3 Summary statistics of data used in this study

| Dataset | Number of input peaks | Total numbers of motif pair with attention attributes |
| --- | --- | --- |
| GATA1_TAL1 | 9193 | 31207433 |
| CTCF_ELF1 | 9811 | 45594624 |
| EGR1_ETV5 | 6923 | 48009238 |
| CEBPA_FOSL2 | 8708 | 20599169 |
| CTCF_EGR1 | 10696 | 73729783 |
| FOSL2_KLF6 | 7919 | 41102238 |
| ETS1_RUNX1 | 12457 | 25143654 |
| CEBPA_SOX6 | 9669 | 25677051 |
| ATF3 | 9474 | 21001351 |
| CEBPB | 6174 | 7945060 |
| SPI1 | 8488 | 12776943 |
| KLA_activated | 11337 | 20371423 |
| KLA_repressed | 17957 | 39830856 |

Supplementary Table 4 Simulation results

| Motif 1 | Motif 2 | Mean p value (N=5) | Standard Error | AUC | PRC |
| --- | --- | --- | --- | --- | --- |
| SPI1 | SIX1 | 0 | 0 | 0.999 | 0.999 |
| SPI1 | CEBPE | 3.43E-286 | 0 | 0.999 | 0.999 |
| RXRA | NRF1 | 0 | 0 | 0.999 | 0.999 |
